# Microelectrode Implantation in Human Insula: Technical Challenges and Recording Insights

**DOI:** 10.3390/brainsci15060550

**Published:** 2025-05-23

**Authors:** Daphné Citherlet, Sami Heymann, Maya Aderka, Katarzyna Jurewicz, B. Suresh Krishna, Manon Robert, Alain Bouthillier, Olivier Boucher, Dang Khoa Nguyen

**Affiliations:** 1 Neurosciences Axis, Centre de Recherche du Centre Hospitalier de l’Université de Montréal (CRCHUM), Montreal, QC H2X 0A9, Canada; daphne.citherlet@umontreal.ca (D.C.);; 2 Department of Neurosciences, Université de Montréal, Montreal, QC H2V 0B3, Canada; 3 Neurosurgery Division, Centre Hospitalier de l’Université de Montréal (CHUM), Montreal, QC H2X 0C1, Canada; 4 Department of Physiology, McGill University, Montreal, QC H0H H9Z, Canada; katarzyna.jurewicz@mcgill.ca (K.J.); suresh.krishna@mcgill.ca (B.S.K.); 5 Department of Psychology, Université de Montréal, Montreal, QC H2V 0B3, Canada; 6 Neurology Division, Centre Hospitalier de l’Université de Montréal (CHUM), Montreal, QC H2X 0C1, Canada

**Keywords:** microelectrode, microcontact, insula, epilepsy, single-unit, multi-unit, spike, Behnke Fried electrode, stereoelectroencephalography, insular implantation

## Abstract

**Background/Objectives**: Intracranial macroelectrode implantation is a pivotal clinical tool in the evaluation of drug-resistant epilepsy, allowing further insights into the localization of the epileptogenic zone and the delineation of eloquent cortical regions through cortical stimulation. Additionally, it provides an avenue to study brain functions by analyzing cerebral responses during neuropsychological paradigms. By combining macroelectrodes with microelectrodes, which allow recording the activity of individual neurons or smaller neural clusters, recordings could provide deeper insights into neuronal microcircuits and the brain’s transitions in epilepsy and contribute to a better understanding of neuropsychological functions. In this study, one or two hybrid macro-micro electrodes were implanted in the anterior-inferior insular region in patients with refractory epilepsy. We report our experience and share some preliminary results; we also provide some recommendations regarding the implantation procedure for hybrid electrodes in the insular cortex. **Methods**: Stereoelectroencephalography was performed in 13 patients, with one or two hybrid macro-microelectrodes positioned in the insular region in each patient. Research neuropsychological paradigms could not be implemented in two patients for clinical reasons. In total, 23 hybrid macro-microelectrodes with eight microcontacts each were implanted, of which 20 were recorded. Spiking activity was detected and assessed using WaveClus3. **Results**: No spiking neural activity was detected in the microcontacts of the first seven patients. After iterative refinement during this process, successful recordings were obtained from 13 microcontacts in the anterior-inferior insula in the last four patients (13/64, 20.3%). Hybrid electrode implantation was uneventful with no complications. Obstacles included the absence of spiking activity signals, unsuccessful microwire dispersion, and the interference of environmental electrical noise in recordings. **Conclusions**: Human microelectrode recording presents a complex array of challenges; however, it holds the potential to facilitate a more comprehensive understanding of individual neuronal attributes and their specific stimulus responses.

## 1. Introduction

Intracranial macroelectrode implantation has proven to be a valuable neurosurgical tool in the clinical assessment of drug-resistant epilepsy. This method helps in the localization of the epileptogenic zone when non-invasive localization techniques are insufficient, and the demarcation of eloquent regions through cortical stimulation [1,2,3]. Intracranial electrode implantations are also great opportunities to investigate cerebral functions in vivo by analyzing brain responses during neuropsychological paradigms [4,5].

Various types of electrodes can be surgically inserted, either individually or in conjunction, such as subdural strip electrodes, grid electrodes, and depth electrodes for stereoelectroencephalography (SEEG). Each type of electrode can contain several macro-contacts, with a size of a few millimeters and capable of measuring neuronal activity from a large population of neurons [6]. In contrast, microelectrodes have a small contact size located at the tips of isolated wires of approximately 10–50 µm diameter. This design facilitates the delineation of extracellular action potentials from neurons [7,8] and enables the recordings of local field potentials (LFP, 1–100 Hz) at a submillimeter scale from compact neuronal clusters, as well as the action potentials emanating from sampled neurons, referred to as multi-unit activities (MUAs). After employing spike sorting techniques, it becomes possible to isolate single-unit activities (SUAs) from these recorded signals [9,10,11,12]. Moreover, microelectrode data are usually sampled at much higher frequencies (up to 40 kHz), allowing the recording of high-frequency oscillations (HFOs, 80–500 Hz). However, it is important to note that due to their high impedance, microelectrodes are more susceptible to artifacts compared to macroelectrodes [12]. The predominant source of interference arises from the electrical recording environment, particularly the electrical line frequency (usually 50/60 Hz). An insufficient signal-to-noise ratio can occur if the electrical environment is excessively noisy, hindering the detection of action potentials. Hybrid depth electrodes, including macro- and microcontacts, seem to exhibit a level of safety and efficacy comparable to that of standard-depth electrodes for intracranial monitoring, offering distinct opportunities to investigate the human brain with single-neuron resolution [13,14,15,16].

Microelectrode recordings have been a significant instrument in exploring the pathophysiology of epilepsy, particularly in understanding ictogenesis. Indeed, microelectrodes implanted within the seizure onset zone (SOZ) can record SUAs during the initiation and progression of seizures, aiding in the characterization of SOZ networks [17,18,19]. Additionally, microelectrodes can detect epileptic markers, including HFOs within the fast ripple band (250–500 Hz) [14,20]. Furthermore, by analyzing the waveform and firing properties of isolated neurons through spike sorting, it becomes possible to differentiate putative pyramidal cells and interneurons, providing insights into their respective contributions to seizure generation [20,21,22]. Finally, microelectrodes have exposed micro-seizures as distinct rhythmic events that would elude detection by macroelectrodes, owing to the enhanced resolution of microelectrodes in sampling the LFPs of significantly smaller neuronal assemblies [18,23,24]. By studying the electrical activity of neurons at a cellular level, clinicians and researchers may gain a deeper understanding of the pathological processes involved in epilepsy and develop more targeted approaches for diagnosis and treatment [25,26].

Currently, microelectrodes are implanted exclusively for research purposes in a small proportion of patients undergoing intracranial EEG evaluation, and have provided an ideal opportunity to record neuronal firing patterns in the human brain during cognitive tasks, leading to important advancements in the understanding of neuropsychological functions, such as speech encoding [27], memory encoding and retrieval [28,29,30], long-term and working memory [31,32,33], emotional processing [34,35], auditory processing [36], and face recognition [37,38]. These findings were obtained notably with microelectrodes implanted in the mesial temporal structures, including the amygdala and hippocampus, as well as in the auditory cortex, the medial frontal cortex, and the occipito-temporal junction.

**Figure 1:**
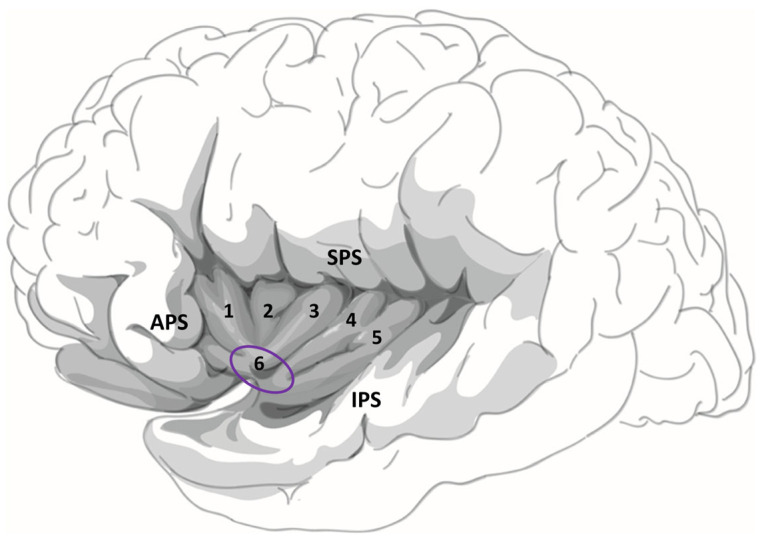
The human insula contains three anterior short gyri (anterior (1), middle (2), and posterior (3)) and two posterior long gyri ((anterior (4) and posterior (5)) separated by the central sulcus of the insula. The most prominent point of the human insula is the apex (6). The insula is delimited by the superior (SPS), inferior (IPS), and anterior (APS) peri-insular sulci. The microelectrodes were implanted in the anterior-inferior part of the insular cortex, as indicated by the purple circle in the figure. Illustration of the insular cortex is provided by Dr. Tram Nguyen. To publish this illustration, permission must be obtained from the copyright owner of the original work.

Over the past decade, studies have demonstrated that a significant number of individuals with drug-resistant epilepsy, who require surgery, have a suspected epileptogenic zone in the insula [1,3,39]. Due to the insula’s deep location within the Sylvian fissure, surrounded by the frontal, parietal, and temporal opercula, and its proximity to other potential epileptic regions, an invasive intracranial implantation is often necessary to confirm the involvement of the insular cortex in epileptic activity [40]. The insula is divided into two parts by the central insular sulcus: the anterior insula (aI) and the posterior insula (pI). The aI consists of three short gyri (anterior, middle, and posterior), while the pI is composed of two long gyri (anterior and posterior) [41] (see Figure 1). The insula has been linked to various functions, encompassing autonomic and vestibular functions, interoception, sensory processing (visceral, somatosensory, auditory, gustatory, and olfactory), as well as affective (emotion and empathy) and cognitive processes (language, attention, memory, and decision-making) [42,43,44,45,46].

Recordings from microelectrodes implanted in the anterior-inferior insular region offer the potential to complement traditional imaging (e.g., functional magnetic resonance imaging) and macroscale electrophysiology (i.e., electrodes with macro-contacts) by adding an additional layer of data to improve our comprehension of the complex role of the insular cortex in emotional processing and interoception. We conducted a pilot study implanting hybrid macro-micro depth electrodes in patients with refractory epilepsy, targeting the anterior-inferior insular region. This deep, anatomically complex brain region presents significant challenges for precise electrode placement and high-quality microelectrode recordings. Nonetheless, its pivotal role in diverse cognitive and affective functions makes it a key target for investigating the neural mechanisms of emotional processing and behavior. The aim of this paper is to share our experiences and findings from insula microelectrode recordings, describe the implantation procedure for the hybrid Behnke–Fried depth electrode, and provide recommendations for improving the quality of microelectrode recordings in this challenging region.

## 2. Materials and Methods

Patients. Thirteen patients (7 females) diagnosed with refractory epilepsy underwent a multimodal evaluation and were recommended for SEEG, including insular sampling, in order to better localize the epileptogenic zone for potential surgical resection. The placement of the electrodes was determined exclusively based on clinical criteria. All patients received explanations and signed a consent form. The study was approved by the CHUM’s ethical committee (#15-018) and was conducted in accordance with the ethical standards laid down in the Declaration of Helsinki. Table 1 provides descriptive characteristics of the study participants. Due to clinical reasons, patients 4 and 11 did not go through the research micro-recording process, and so only 20 out of the 23 implanted electrodes were recorded.

**Figure 2:**
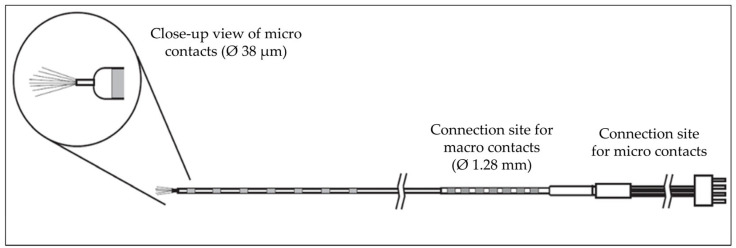
Schema of the Behnke–Fried electrode.

Electrophysiology. The number of macroelectrodes implanted in each patient ranged from 10 to 17, including 1 or 2 hybrid macro-microelectrodes (see Table 1). Clinical iEEG macroelectrode recordings were acquired using a Nihon-Kohden long-term monitoring system (Nihon-Kohden System, Tokyo, Japan). All macroelectrode signals were acquired continuously with Nihon-Kohden Headstages (Nihon-Kohden, Neurofax Mini Flat Junction Box, Tokyo, Japan). Microelectrode signals were recorded using a 32-channel NeuroPort system (Blackrock Microsystems, Salt Lake City, UT, USA), with a 16-channel Cabrio Headbox connector (Ad-Tech Medical, Racine, WI, USA). For microelectrode recordings, we used the hybrid Behnke–Fried (BF) depth electrode (Ad-Tech Medical, Racine, WI, USA) (see Figure 2), which can record deep structures like the insula. The electrode consists of an 8-contact macro component (90-10 platinum-iridium, outer diameter 1.28 mm, length 1.57 mm) and a 9-contact micro component (90-10 platinum-iridium, outer diameter 38 μm). The micro component is covered by a protective sheath and runs through the lumen of the macro component. The macro component was used for the standard clinical recording, and the micro component was used for the research recording. Eight of its microwires acted as the active recording electrodes and the 9th acted as a reference (by default but may be changed manually). The microelectrode tails were connected to the Cabrio connector. Data were recorded at 30 kHz during the experimental tasks exclusively (i.e., a few hours per day). The signal from the electrodes was high-pass filtered at 250 Hz. The implantation of hybrid electrodes proceeded without any complications.

As the procedure progressed, several materials were replaced, such as the Cabrio headbox connector (from patient #2 onward), the shielding of all cables (from patient #8 onward), and a preamplifier (from patient #9 and onward).

**Figure 3:**
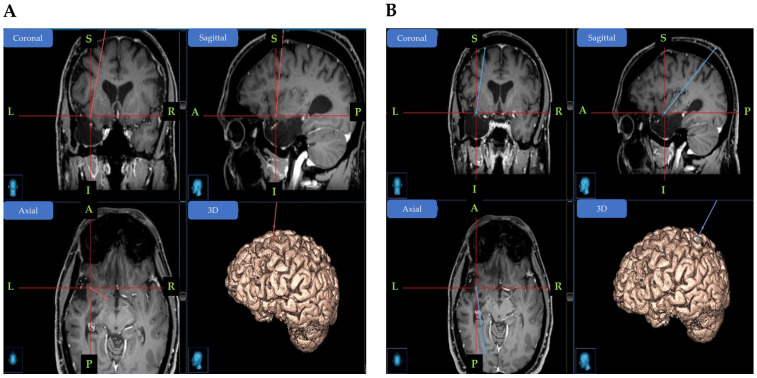
Example of the preoperative planning. (**A**) Hybrid electrode implanted in the left anterior insula (anterior trajectory), and (**B**) Hybrid electrode implanted in the left posterior insula (posterior trajectory). R: right; L; left; A: anterior; P: posterior; S: superior; I: inferior.

Preoperative planning. All patients underwent a 3T brain MRI with 3D T1 + GAD and FLAIR protocol. The planning was conducted on a StealthStation, Medtronic Inc. (Louisville, CO, USA) Planning station. We used two optional trajectories toward the insula for single unit recording: an anterior trajectory that passes through the superior frontal gyrus and a posterior trajectory, parallel to the long insular gyri entering through the superior parietal lobule (Figure 3). Both trajectories ended in the antero-inferior corner of the insula. Fine trajectory adjustments were performed to assure a safe distance from blood vessels. Because microwires protrude beyond the distal end of the macroelectrode, we made sure there was 5 mm of insular cortex post-target as well.

Operative procedure. The patient was brought into the operating room (OR) where they underwent general anesthesia and endotracheal intubation. The patient’s head was completely shaved, and local anesthesia (xylazine and bupivacaine) was injected into the four skull pin sites. A stereotactic frame (CRW, Integra Inc., Tokyo, Japan) was placed, and the patient was transferred to the computed tomography (CT) scan suit. A stereotactic localizer was placed on the frame, and the patient underwent a head CT scan (axial images, 1 mm slice thickness). The localizer was removed, and the patient was taken back to the OR. The scan images were transferred to the planning computer and merged with the previously acquired MRI images. The x, y, z coordinates and the trajectory angles for each of the targets were defined. The bone thickness was calculated on the planning computer for each of the trajectories.

The patient was positioned supine on the operating table with the CRW frame fixed to the Mayfield head holder. Their head was entirely prepped with antiseptic solution and covered by an incise drape. The stereotactic head frame was assembled on the CRW base ring and secured with three locking knobs. Two sterile drapes were draped around the head frame to create a sterile field. The coordinates and angles were set on the stereotactic frame and double-checked by two neurosurgeons. A reducing tube was inserted through a guide block on the frame, and the exact entry point on the skin was marked. A 0.5 cm skin incision was performed down to the skull, and hemostasis was achieved using bipolar cautery if needed.

A twist drill was inserted through the reducing tube down to the skull. A drill stopper was set to a distance of 5 mm plus the calculated bone thickness, in order to ensure piercing the dura matter. A hex wrench with anchor bolt was inserted through the reducing tube into the drilled hole. The anchor bolt was screwed until a significant increase in resistance was felt. The distance between the target and the external edge of the guide block was 170 mm. The length of the k-wire was measured on a metal ruler and marked at 150 mm from its distal tip. This causes the k-wire to stop 20 mm before the target to minimize the risk of deep vascular injury. The macro component of the BF electrode was measured as well and marked at 170 mm from its distal tip. The microwires at the distal tip of the BF micro component were cut diagonally to a length of 3–5 mm using sharp scissors. The microwires were separated from each other using gentle finger movements. The green sheath was pulled over the microwires. The K-wire was slowly inserted through the anchor bolt until the marked point aligned with the external part of the block guide, and then pulled back. The assembled micro-macro BF electrode was then inserted in the same way until reaching the marked depth. The electrode stylet was removed, and the anchor bolt screw tightened. The same procedure was repeated for the rest of the electrodes. The entire head was cleaned and the frame removed. A full head dressing was applied, and the patient was awakened and taken to the recovery room. After a night of observation in an intermediate care unit, a 3D T1 + FLAIR head MRI was performed, and the patient was transferred to the EEG monitoring unit.

Post-implant monitoring. The anatomical locations of the electrodes were routinely verified after the implantation by MRI coregistered to the preoperative MRI. Special attention was taken to ensure that the distal end of the metallic artifact was located in the insular cortex. In addition, by advancing the marker on the planning station a few millimeters further on the same trajectory, we confirmed the presence of cortex where the microwires were supposed to be located. Following implantation, the electrodes were carefully covered with a bandage, forming a ’headwrap’, ensuring their protection.

Experimental sessions. Patients were tested in their hospital room as soon as possible after SEEG implantation (an average of 3 days after surgical implantation). They were connected to both macro and microelectrode acquisition systems during the experimental tasks. A ground electrode was placed on the left shoulder of each patient. During the first tasks, patients sat in a chair facing a laptop on which pictures were presented. In the first task, we presented pictures selected from the Nencki Affective Picture System (NAPS [47]), a standardized set of emotion-eliciting color pictures (i.e., neutral, happiness, sadness, and disgust). The total task consisted of five blocks, totaling 300 stimuli, presented in a pseudorandomized order over 2000 ms with a random inter-stimulus interval (ISI) ranging from 500 to 2000 ms. Each block comprised a set of 60 pictures. The second task mirrored the first, with the additional instruction for the patient to indicate the emotion elicited by each picture. The third task involved face emotion pictures, following the same parameters as the previous tasks. In the fourth task, patients were engaged in a three-minute interoceptive task. They were first instructed to recall and re-experience pleasant personal life events that had occurred throughout their life, such as moments of success, personal achievements, and family gatherings, and to be mindful of their bodily sensations. Subsequently, patients were instructed to recall and re-experience negative life events for 3 min. For the last two patients, we introduced two additional tasks: (1). The patients were instructed to generate highly enjoyable food imagery internally (i.e., self-generated) for 3 min; (2). The patients participated in passive listening sessions involving both pleasant and unpleasant music; each session lasted 3 min.

Electrode removal. After completing the monitoring period, the electrodes were removed. If immediate resective surgery was planned, the electrodes were removed under general anesthesia just prior to surgery. Otherwise, the electrodes were removed at bedside in the EEG monitoring unit. After meticulous disinfection of the electrode entry sites, the anchor bolts were released, and the electrodes were gently pulled out. A surgical staple was placed at each electrode site to prevent CSF leakage through the skin incision. In our series of 13 patients, no postoperative complications related to electrode implantation were observed.

Spike extraction and experimental analysis. Potential epileptic discharges were screened, and no epileptiform activity was found in our recordings. For the 4 patients from whom we obtained usable microcontact data, spike detection and sorting were performed on each microcontact separately using the WaveClus3 (Version 3.0.3) open source spike sorting tool [48], which is well-established and commonly used in the field. Within WaveClus3, each channel’s signal (recorded at 30 kHz sampling rate) first goes through a second-order elliptic filter ranging between 300 Hz and 3000 Hz, with the low-frequency and high-frequency cutoffs chosen to reduce low-frequency drift and high-frequency noise, respectively, while maintaining the signal in the range relevant for spikes. Next, spikes are detected using an amplitude threshold, defined as a multiple of the recording’s noise level, and a brief refractory period, assuring each spike is only detected once. These parameters can be changed manually, but we used the default values of a multiple of 5 for the amplitude threshold and the default refractory period, set as 1.5 ms. Based on visual verification of our spike sample, we used a threshold type of “neg”, meaning only negative crossings of the threshold are considered for the detection.

After their detection, the goal is to assign each detected spike to one of a few clusters consisting of spikes either from an isolated single neuron or from a small group of multiple neurons. For this, the detected spikes are transformed using wavelet decomposition to compactly represent their structural and temporal features while suppressing noise. Then, using Superparamagnetic Clustering (SPC) [49], the spikes are grouped based on these features, such that each cluster is regarded as originating from a distinct unit or multi-unit group. This idea relies on the principle that a recorded spike’s shape and temporal properties are mostly influenced by the structural and spatial properties of its source [50]. SPC is an unsupervised process derived from the field of statistical mechanics. It groups data points based on their pairwise similarities according to a “temperature” variable, which determines the similarity level required to group data points to the same cluster. The higher the temperature is, the lower the similarity required between data points to belong to the same cluster. The SPC process is performed with different temperature values, and the final clusters are chosen based on a threshold that considers how their size changes as the temperature changes.

## 3. Results

Table 1 provides a summary of the recordings. In total, 20 hybrid macro-microelectrodes were implanted and recorded from in the anterior-inferior insular region across 11 patients with refractory epilepsy, totaling 160 microcontacts (eight microcontacts per electrode). In the first seven patients, the recorded data were compromised by 60 Hz line-noise contamination and lacked detectable neuronal activity.

**Figure 4:**
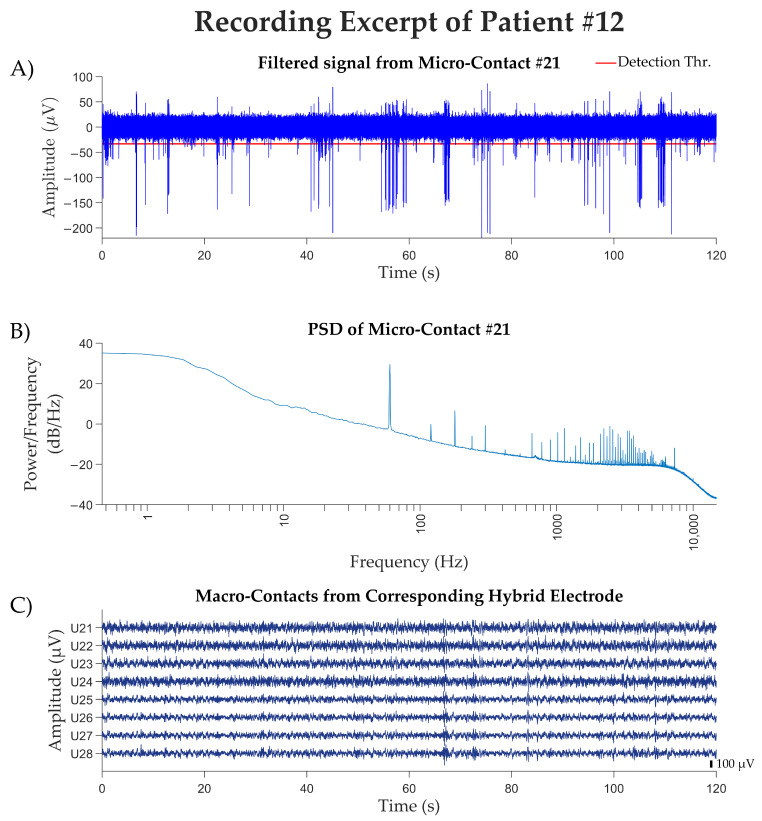
Recording excerpt from patient #12. (**A**) A 120 s segment from one of the patient’s micro-contacts, with the signal presented after being filtered with a 300 Hz–3000 Hz elliptic filter. The spike detection threshold used by WaveClus3 is marked with a red line. (**B**) The power spectral density of the signal from the entire session (14 min, 39 s, prior to elliptic filtering) recorded by the micro-contact. The PSD was computed using MatLab’s (2024A) pwelch method with a Hamming window of 65,536 samples, 50% overlap, and an FFT length of 65,536 points. (**C**) The SEEG local field potentials recorded from the eight macro-contacts of hybrid electrode U2, which includes microcontact #21. Signals were band-pass filtered between 0.5 and 300 Hz, with a notch filter applied at 50 Hz, using implementations from the MNE-Python package (mne: 1.7.1). Anatomically, U21 is the deepest contact located within the anterior insula, U26 is located at the outer edge of the anterior insula, U27 is located within the white matter, and U28 is located outside the insula.

**Figure 5:**
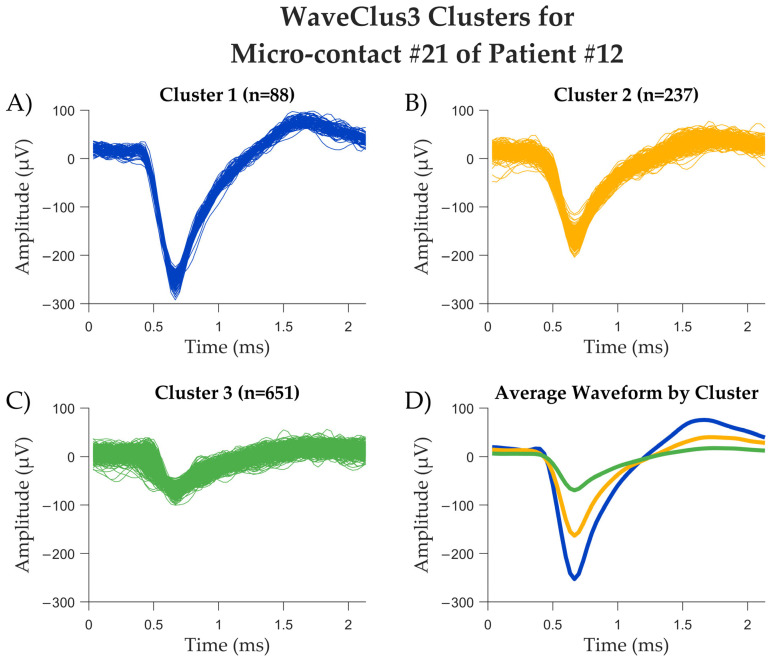
Analysis results from one of patient 12’s micro-contacts. (**A**–**C**) The spikes detected in the recording, as divided into three clusters by WaveClus3. The number of spikes assigned to each cluster is provided in the title as n. (**D**) The average spike shapes of each cluster, by their corresponding color (blue for cluster #1, yellow for cluster #2, green for cluster #3).

In the final four recorded patients, after equipment replacement and improved shielding, the 60 Hz artifact was no longer an issue. We recorded activity from 64 microcontacts (channels) across eight electrodes from these patients. By visual inspection, we detected spiking activity in 13 of these channels (13/64 = 20.3%; Table 2). We then passed the signal from each channel to WaveClus3, with a negative detection threshold setting of five times the standard deviation. We isolated 12 clusters that correspond to the putative single-unit activity of 12 separate neurons, and 21 additional clusters corresponding to the putative multi-unit activity of 21 multi-neuron groups. Figure 4 shows the electrophysiological recordings from patient #12, including a 120-s recorded from microcontact #21, the power spectral density of the micro-contact #21, computed across the entire recording session, and the eight macro-contacts of the same hybrid electrode (U2) that includes micro-contact #21. Figure 5 shows an example of the spikes detected in one of the microcontacts, separated into clusters using the WaveClus3 algorithm. In this particular contact, three distinct single units were obtained.

**Figure 6:**
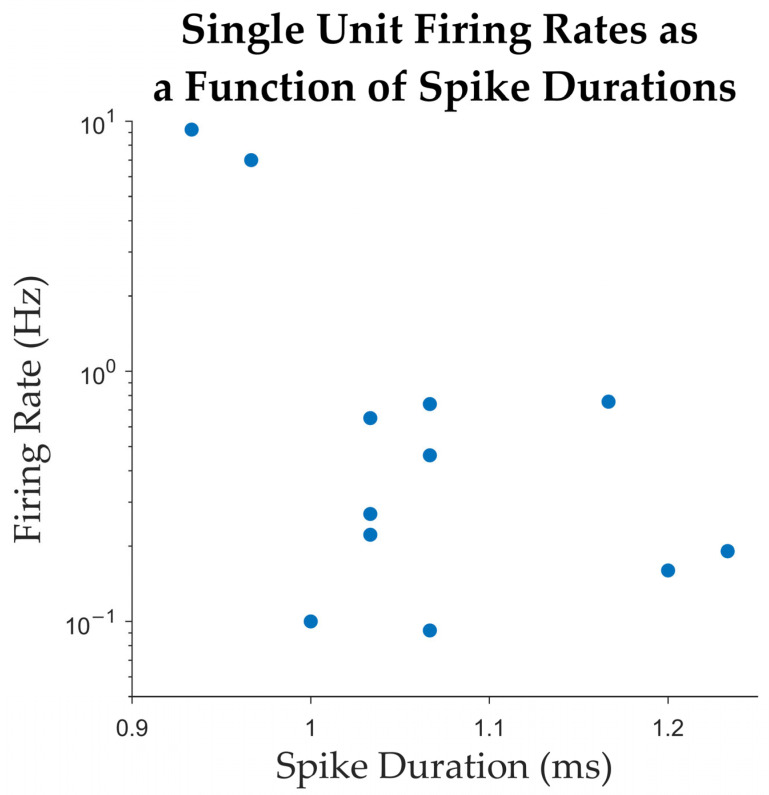
Average firing rates plotted against spike durations. Each dot in the plot corresponds to one single unit detected.

**Figure 7:**
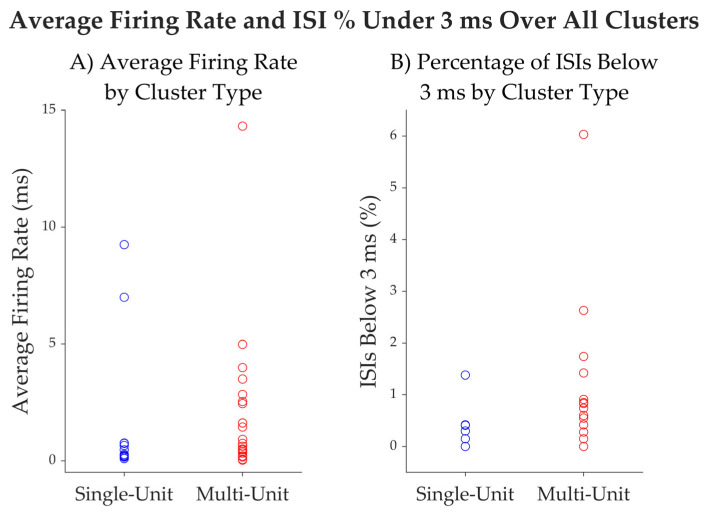
(**A**) Average firing rate of clusters, separated into single-unit and multi-unit clusters. (**B**) The percentage of ISIs below 3 ms for each cluster, separated into single-unit and multi-unit clusters.

**Figure 8:**
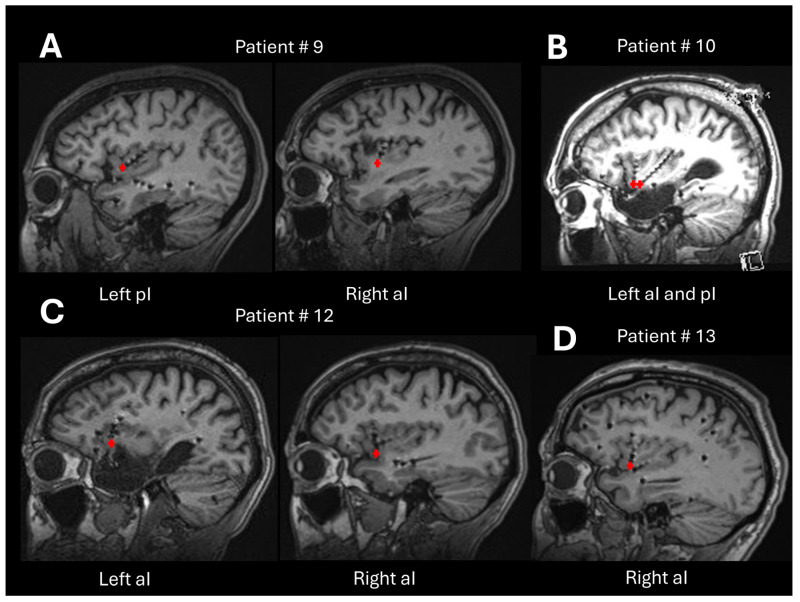
(**A**–**D**) Post-implantation sagittal MRI slices from the patients enrolled in the study (patients #9, #10, #12, and #13). Red crosses indicate the exact locations of the microcontacts of the hybrid electrodes. In all cases, the microcontacts are located within the antero-inferior portion of the insular cortex.

We characterized single units using standard metrics [51] and tabulate them in Table 3. Across all single units, the average spike width, measured as the trough-to-peak duration, was 1.07 ± 0.09 ms (mean ± standard deviation; Figure 6). The peak signal-to-noise ratio (SNR), calculated as the ratio between the trough-to-peak amplitude and the standard deviation of the background noise (as estimated by WaveClus [52]) was 5.56 ± 2.65. The mean firing rate across all single units was 1.66 ± 3.07 Hz. The percentage of inter spike intervals (ISIs) that were lower than 3 ms was 0.22 ± 0.4%, with a median of 0%. These firing-rate and ISI percentage (below 3 ms) values are plotted along with those for multi-units in Figure 7. The mean modified coefficient of variation in the ISI (commonly referred to as CV2 [53]) was 1.1 ± 0.14, and not significantly different from 1 (the value for a Poisson process). Figure 8 illustrates the post-implantation MRI slices showing the micro-contact locations for patients #9, #10, #12, and #13. In all cases, the microcontacts were located within the antero-inferior portion of the insular cortex, as confirmed by anatomical visualization.

## 4. Discussion

As anticipated, the endeavor to obtain unit recordings and local field potentials using microelectrodes in the clinical environment presented certain challenges. These challenges encompassed the absence of discernible neuronal activity signals, unsuccessful dispersion of microwires, and contamination of recordings due to environmental electrical noise. After several attempts, successful recordings of neural activity from isolated neurons were accomplished during our neuropsychological tasks.

Following the implantation of hybrid macro-microelectrodes, the recordings during experimental tasks posed inherent challenges that needed to be addressed. Obstacles encompassed the absence of neuronal activity signals, interference of environmental electrical noise, artifacts caused by the reference and ground choices, and unsuccessful microwire dispersion.

### 4.1. Absence of Neuronal Activity Signals

During the patients’ stay for epilepsy unit monitoring, we encountered limitations in recording the complete neuronal activity using our Cabrio headbox 16-channel system. Despite having implanted electrodes in the relevant regions, certain channels exhibited a constant lack of detectable neural signal. We propose that this issue may have resulted from breaks in the microwires caused by excessive tensile forces during various physical manipulations, such as headwrap changes, surgery, initial hook-up, and so on. Moreover, it is important to note that placing microwires in a region with a thin layer of grey matter (usually less than 5 mm) such as the insula can result in a lower yield of recorded neurons [54]. Despite technically successful implantations without any observed failure modes, certain microwires failed to record any neuronal units. We postulate that these specific microwires were not positioned in close proximity to active cells. Furthermore, although electrochemical impedance spectroscopy (EIS) over a broad frequency range was not performed in this study, future investigations using EIS could provide valuable insight into frequency-dependent impedance stability and its relationship to signal fidelity, particularly in hybrid micro-macroelectrode configurations.

### 4.2. Power Line Interference Issue

Power line contamination is a term used to describe electromagnetic interference that occurs at frequencies of 50/60 Hz, which originates from power lines or external sources of voltage. This interference generates an electric field with the capability to produce coupled currents in conductors that lack shielding. As a consequence, this leads to the occurrence of an extra alternating voltage in recorded data [55]. In a hospital setting, it is crucial to acknowledge that various types of equipment and personal electronic devices can emit electromagnetic radiation (e.g., beds, chair, lights, monitors, cables). Furthermore, it is important to recognize that both the patient and the components of the recording system, including the electrodes and connecting cables, can function as antennas in this environment. During our initial recordings, we frequently encountered data contamination caused by electromagnetic interference. As a result, we did not record the data when there was clear line noise contamination and no detectable spiking activity. To address this issue, we implemented shielding measures for the cable connecting the 16-channel headstages to the amplifier, employing aluminum foil to create a shielding effect similar to a Faraday cage around the recording cables. The implementation of this shielding technique significantly improved the quality of our data. Nevertheless, even when operating on battery power, a laptop can introduce interference for the microelectrodes if the patient interacts with the keyboard (it was the case during our active experimental tasks). A viable solution, particularly for cognitive tasks that require responses, involves employing optical cables to connect to button response boxes, or wireless keyboards and/or mice, as suggested by Lehongre and colleagues [56].

### 4.3. Artifacts: Reference and Ground

The choice of reference electrode for microelectrode recordings is a crucial consideration. This reference electrode serves as a baseline for measuring the electrical activity of the target neurons. It should ideally have a stable and low impedance, share as much of the common noise in the signal electrodes as possible, as well as have a minimal independent contribution to the overall noise in the recording [54]. When selecting the reference electrode for microcontact recordings, several factors need to be considered. Firstly, one important factor is the proximity of the reference electrode to the recording site (i.e., to have the reference electrode as close as possible to the target neurons to minimize the introduction of additional noise). Secondly, one needs to consider the type of reference electrode used. In general, common choices include large surface electrodes (i.e., applied to the skin of the subject), microelectrodes, or a combination of both. Large surface electrodes provide a low-impedance reference but may introduce additional noise due to their larger size. On the other hand, microcontacts can match the geometry and impedance of the recording sites but may have higher impedance and contribute more thermal noise to the recordings [8,57,58]. In our experiments, we made several attempts to mitigate artifacts caused by the selection of the reference electrode. From our seventh recorded patient, it was determined that using the ninth microwire of our microelectrode was a more advantageous option compared to using an electrode on the mastoid. This optimization of the reference electrode placement significantly enhanced the quality of our microelectrode recordings afterwards. On the other hand, the ground electrode was strategically placed in a neutral region, specifically on the shoulder of our patients, to ensure a significant distance from any potential electrical interference sources.

### 4.4. Unsuccessful Microwire Dispersion

**Figure 9:**
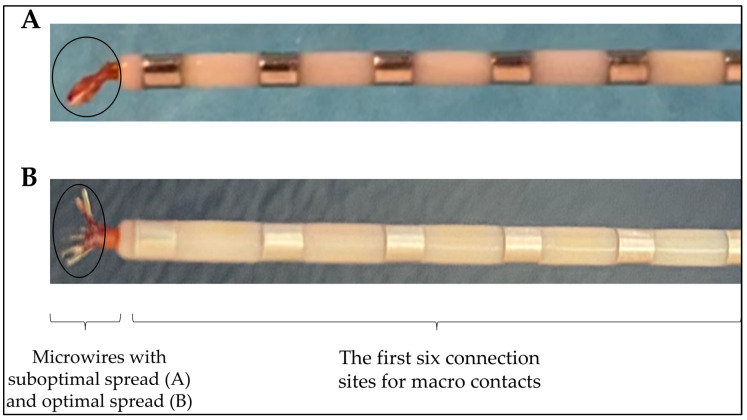
Microwires of Behnke–Fried electrodes: (**A**) suboptimal; (**B**) optimal.

Our post-implantation imaging analysis revealed that some microelectrodes were in grey matter as expected, but some were in white matter, partly explaining our difficulty to record neuronal activity. Moreover, in our first two cases, we noticed that in some BF electrodes, the microwires were stuck together as a bundle. As this can be a cause for recording failure, we adjusted the implantation method and made sure to spread the wires with gentle finger movement before inserting them. Figure 9 shows suboptimal (A) and optimal (B) wires spread as seen post removal.

### 4.5. Successful Recordings

After troubleshooting over the first seven unsuccessful recording processes, our procedures reliably yielded successful recordings of both single neurons and multi-unit clusters in the most recent four patients in our series. We have outlined our troubleshooting steps above so that they can be helpful to guide similar efforts by others. The statistics of our isolated single-units from the insula roughly match those reported in the literature for similar recordings from the human hippocampus, despite using different spike detection methods [51]. Our ongoing recordings will enable more detailed characterization of the properties of insular neurons in the context of various neuropsychological tasks and also enable more detailed comparisons with other datasets as well as our own recordings from non-insular areas. The main recommendations are presented in Table 4 as a guide.

## 5. Conclusions

This study showed the feasibility of recording insular microelectrode signals in vivo in patients with epilepsy. We provide detailed technical guidelines to enable the successful implantation and the recording of hybrid macro-microelectrodes in the human insular cortex. Although recording from the human insular cortex using microelectrodes presents a complex array of challenges, this methodology holds significant promise for advancing our understanding of the role and functions of specific insular subregions. To our knowledge, very few studies have reported physiological single- and multi-unit activities from the human insular cortex using microelectrodes during awake cognitive and affective tasks. The insula, a deep and anatomically complex region, has largely remained unexplored at this scale of resolution in humans. Our findings provide rare insights into the dynamics of insular activity under non-ictal conditions and highlight the potential of microelectrode recordings to study deep brain structures in the context of human cognition and emotion.

## Figures and Tables

**Table 1:** List of study participants and localization of implanted electrodes.

Patient Number	Sex	No. of Electrodes Implanted	Localization of Electrodes Implanted	No. of Hybrid Depth Electrodes and Localization ^1^	Surgery Following Implantation
1	F	17	8 R (aI, pI, amygdala, aH, pH, PCC, precuneus, cuneus) and 9 L (aI, pI, amygdala, aH, pH, PCC, precuneus/cuneus)	2 (R pI, L pI)	L temporal lobectomy
2	M	12	6 R (aI, pI, amygdala, aH, pH, OT, O) and 6 L (aI, pI, amygdala, aH, pH, OT, O)	2 (L and R aI/pI)	No surgery
3	F	14	2 R (pI, aI) and 12 L (pI, aI, P, angular, T, F, PCC)	1 (R pI)	No surgery
4	M	10	10 R (aI, pI, aH, pH, amygdala, OF, F op)	1 (R pI)	No surgery
5	M	10	10 R (aI, pI, amygdala, aH, pH, OF, ACC, PCC, median F)	2 (R aI, R pI)	No surgery
6	F	13	4 R (ACC, mesial F, OF) and 9 L (amygdala, aH, pH, OF, ACC, MCC, median F)	2 (L aI, L pI)	L temporal lobectomy
7	F	12	12 R (pI, P, ant and post precuneus, PCC, ACC, MCC)	1 (R pI)	No surgery
8	F	12	12 R (aI, pI, amygdala, pH, aH, ACC, IFG, OF, F P T op, P)	2 (R aI, R pI)	R anterior temporal lobectomy
9	F	11	1 R (aI) and 10 L (aI, pI, amygdala, aH, pH, OF, T)	2 (R aI, L pI)	No surgery
10	M	10	10 L (aI, pI, p parahippocampal, op T, fusiform, OF)	2 (L aI, L pI)	L insular lobe resection
11	M	10	10 R (aI, pI, ACC, MCC, PCC, OF, median F)	2 (R aI, R pI)	No surgery
12	F	14	8 R (aI, superior insula, T, OF, PCC, op P-T) and 6 L (aI, pI, amygdala, aH, pH, OF)	2 (R aI, L aI)	No surgery
13	M	16	16 R (aI, pI, amygdala, F op, P op, ACC, PCC, ant/mid/post median frontal, fusiform area)	2 (R aI, R amygdala)	R frontal corticectomy
Total		161		23	

R right, L left, aH, anterior hippocampus, pH posterior hippocampus, ACC anterior cingulate cortex, PCC posterior cingulate cortex, MCC middle cingulate cortex, F frontal, P parietal, O occipital, T temporal, OT occipito-temporal, OF orbitofrontal, aI anterior insula, pI posterior insula, IFG inferior frontal gyrus, op operculum. ^1^ All micro contacts were localized in the anterior-inferior insula and were implanted by SEEG.

**Table 2:** Summary of single-unit clusters and putative multi-unit clusters for each of the last four recorded participants.

Patients ^1^	Duration of Recording (min:s)	No. of Microcontacts ^2^ with Neuronal Activity	No. of Well Isolated Units	No. of Putative Multi Units Detected
9	05:08	2/16	1	3
10	24:25	6/16	6	9
12	14:40	3/16	4	6
13	03:57	2/16	1	3
		13/64 (20.3%)	12	21

^1^ Patient #11 not recorded. ^2^ All micro contacts were localized in the anterior-inferior insula and were implanted by SEEG.

**Table 3:** Metrics for each well-isolated single unit.

Unit ID	Average Firing Rate (Hz)	Average Spike Width (ms)	% Intervals Below 3 ms	Trough to Peak SNR	CV2
1	0.22	1.03	0	5.87	1.1
2	9.24	0.93	1.38%	3.82	1
3	6.99	0.97	0.41%	4.32	0.8
4	0.65	1.03	0	5.62	1
5	0.16	1.20	0	7.33	1.2
6	0.19	1.23	0	3.87	1.3
7	0.09	1.07	0	6.22	1.2
8	0.76	1.17	0.30%	2.93	1.1
9	0.27	1.03	0.42%	7.64	1.2
10	0.74	1.07	0.15%	3.27	1.1
11	0.1	1.00	0	12.33	1.2
12	0.46	1.07	0	3.55	1.1
mean	1.66	1.07	0.22	5.56	1.10
median	0.3	1.05	0.00	4.97	1.1
SD	3.07	0.09	0.00	2.65	0.14

**Table 4:** Summary of guidelines.

	Encountered Difficulties	Recommendations
Implantation surgery	Microwires bundled	Spread the wires before insertion. Check the spread wires in the MRI post-implantation.
	Hybrid electrode trajectories	Prior to the insertion of microelectrodes into the macroelectrodes, microwires must be cut to the desired length defined during the planning surgery.
Post- implantation surgery	Hybrid electrodes without microcontact signal	Do not bend and/or pull the cables of the electrodes. Test the patient as soon as possible following the surgery implantation. Confirm the localization of the microcontacts in the grey or white matter.
Testing	Power line contamination	Use shielded cables, aluminum foils. Unplug all devices in the room of the patient. Use a power conditioner for laptop, optical cable, and/or wireless. No cellphone in the room.
	Artifacts	Check the reference electrode and the ground. Stabilize the Cabrio connector, headstage, and cables. Minimize movements by the patient. All connector cables in the same direction.

## Data Availability

Dataset available on request from the authors. The data are not publicly available due to ethical reasons.
